# Hysteresis in As-Synthesized MoS_2_ Transistors: Origin and Sensing Perspectives

**DOI:** 10.3390/mi12060646

**Published:** 2021-05-31

**Authors:** Carlos Marquez, Norberto Salazar, Farzan Gity, Jose C. Galdon, Carlos Navarro, Carlos Sampedro, Paul K. Hurley, Edward Yi Chang, Francisco Gamiz

**Affiliations:** 1 Nanoelectronics Research Group (CITIC-UGR), Department of Electronics, University of Granada, 18071 Granada, Spain; jcgaldon@ugr.es (J.C.G.); carlosnm@ugr.es (C.N.); csampe@ugr.es (C.S.); fgamiz@ugr.es (F.G.); 2 Nanoelectronic Materials and Devices Group, Tyndall National Institute, University College Cork, T12 R5CP Cork, Ireland; farzan.gity@tyndall.ie (F.G.); paul.hurley@tyndall.ie (P.K.H.); 3 International College of Semiconductor Technology, National Yang Ming Chiao Tung University, Hsinchu 30010, Taiwan; edc@mail.nctu.edu.tw

**Keywords:** two-dimensional materials, light sensor, molybdenum disulfide, MoS2, trapping, reliability

## Abstract

Two-dimensional materials, including molybdenum disulfide (MoS${}_{2}$), present promising sensing and detecting capabilities thanks to their extreme sensitivity to changes in the environment. Their reduced thickness also facilitates the electrostatic control of the channel and opens the door to flexible electronic applications. However, these materials still exhibit integration difficulties with complementary-MOS standardized processes and methods. The device reliability is compromised by gate insulator selection and the quality of the metal/semiconductor and semiconductor/insulator interfaces. Despite some improvements regarding mobility, hysteresis and Schottky barriers having been reported thanks to metal engineering, vertically stacked heterostructures with compatible thin-layers (such as hexagonal boron nitride or device encapsulation) variability is still an important constraint to sensor performance. In this work, we fabricated and extensively characterized the reliability of as-synthesized back-gated MoS${}_{2}$ transistors. Under atmospheric and room-temperature conditions, these devices present a wide electrical hysteresis (up to 5 volts) in their transfer characteristics. However, their performance is highly influenced by the temperature, light and pressure conditions. The singular signature in the time response of the devices points to adsorbates and contaminants inducing mobile charges and trapping/detrapping carrier phenomena as the mechanisms responsible for time-dependent current degradation. Far from being only a reliability issue, we demonstrated a method to exploit this device response to perform light, temperature and/or pressure sensors in as-synthesized devices. Two orders of magnitude drain current level differences were demonstrated by comparing device operation under light and dark conditions while a factor up to 10${}^{5}$ is observed at vacuum versus atmospheric pressure environments.

## 1. Introduction

In the last decades, the limitations in chip integration to achieve Moore’s law density requirements have been overcome through the progressive scaling of the transistor dimensions (particularly channel length) [1,2], the incorporation of high-κ insulators [3] and the design of new device architectures (FinFet, SOI, trigate) [4] to achieve better control of the device performance. The advances in the lithography (deep ultraviolet (DUV) and multi-patterning techniques) have been employed to shrink the dimensions of transistors down-to sub-10 nm channel lengths [2]. However, to support more scaled process technologies, the half-pitch resolution should be improved. In this context, extreme ultraviolet (EUV) lithography has been proposed assuming that costly new manufacturing equipment will be needed [5]. At the same time, other alternatives to increase the performance of the integrated circuits have been considered.

Silicon channel material substitution has been one of the most studied options to address Moore’s law [6]. On the one hand, III-V and Germanium (Ge) compounds have been widely analyzed with outstanding results in high power, memory and photonics applications [7,8]. However, limitations in any of the carrier mobilities (electrons or holes), wider bandgaps and difficulties in their integration into silicon processes have limited the applications in complimentary MOS (CMOS) technology [6]. On the other hand, two-dimensional materials, led by the discovery of graphene [9,10], have attracted tremendous attention due to their promising electrical properties [5,11]. Thanks to their low dimensionality, these thin materials present an optimal electrostatic control of the channel [12], flexibility and extremely sensitive capabilities to the changes in their surroundings. Despite the fact that graphene seems to be less attractive to the logical electronic applications due to its zero bandgap, other two-dimensional materials, such as transition metal dichalcogenides (TMDs), have demonstrated an indirect to direct bandgap modulation depending on the layer thickness inside an interesting range for electronic applications [13]. Within the family of TMDs, MoS${}_{2}$ and WS${}_{2}$ with thickness-dependent bandgaps in the range of 1.2–1.8 eV and usually n-type behavior have received special attention [14].

Intermediate solutions between the complete replacement of silicon by alternative materials and silicon hegemony have been proposed. Three-dimensional (3D) integration consists of employing multiple vertically stacked active layers (containing active devices such as transistors, diodes and logic memory devices) to achieve higher integration density, lower power consumption and better signal integrity [15,16]. In this context, the incorporation of atomically thin 2D-material-based devices and interconnects in monolithic-3D integration have been already proposed to overcome several major problems of conventional technologies such as inter-tier signal delay, chip overheating and inter-tier electrical interference problems [17,18]. In fact, some advances have already been demonstrated using 2D materials as back-end of the line (BEOL) to improve the interconnect scaling [19] or the chip heat dissipation [18]. Additionally, the 3D integration of 2D devices to perform logic memory cells [20], opto-electronic detectors or sensors [21,22], are promising approaches to realize system-on-chip (SoC) designs solving the density constraints. In this context, the integration of 2D materials could seem particularly interesting in sensing applications due to their atomic thickness and extreme sensitivity to changes in the environment. Nonetheless, there are still technological limitations regarding the two-dimensional material integration in semiconductor fabrication flow [23]: (i) the majority of studies that explore TMDs obtain films using methods that are not scalable, such as mechanical exfoliation or methods that are not CMOS compatible such as synthesis at high temperatures (T > 450 °C); (ii) the devices rarely accomplish the promised theoretical properties of these 2D materials. Defects and impurities inducing Fermi level pinning at the metal interfaces, Schottky-barrier formation, current hysteresis or Coulomb scattering are some of the reliability issues of the fabricated 2D devices [24,25,26,27,28]. Despite some performance and reliability improvements having been achieved by employing interfacial compatible thin insulator such as hexagonal boron nitride [29], vertically stacked heterostructures [30] or high-quality encapsulation processing [26], these processes may limit the environmental sensing capabilities of these two-dimensional devices. Regrettably, without these encapsulation or passivation processes, the selectivity and performance of 2D materials rapidly decreases as defects, imperfections and dislocations in the material and at the interfaces may mask the response to the detection target [31]. In spite of this difficulty, at the same time, defects and imperfections can also lead to electrical responses that could be used to enhance the sensing capabilities of these devices. Residual ions, adsorbates and trapped carriers are translated to mobile and fixed charges. These charge-related phenomena are also affected by device surroundings such as temperature, humidity or light conditions, which means that, if controlled and characterized, they could be used as sensing parameters.

In this work, our goal is to investigate the disturbing mechanisms that affect the operation of as-synthesized MoS${}_{2}$ back-gated devices under different work conditions and evaluate how these mechanisms can be employed to enhance the sensing and detecting capabilities of the fabricated devices. The accomplishment of sensors employing as-synthesized two-dimensional materials without the need of passivation or encapsulation processes could lead to a huge advance in the integration of these thin materials and the relaxation of the fabrication complexity.

## 2. Materials and Methods

**Figure 1:**
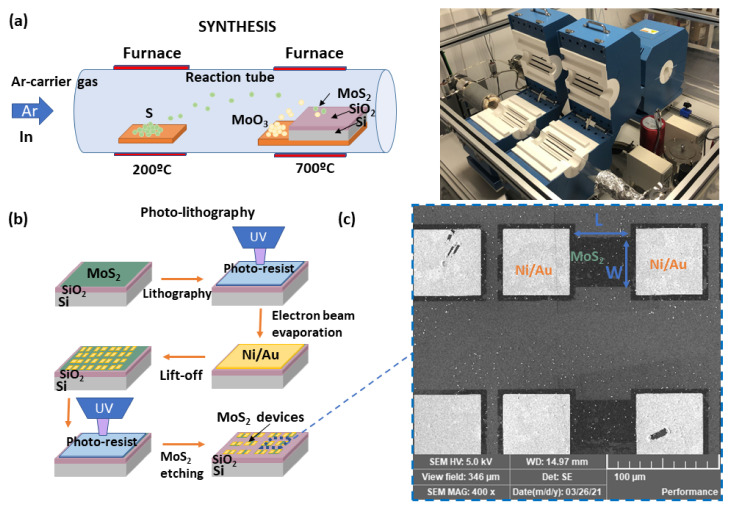
(**a**) Scheme (**left**) and picture (**right**) of the chemical vapor deposition synthesis carried out in a two-temperature-zone tube furnace. Sulfur (S) and molybdenum trioxide (MoO${}_{3}$) are placed separately. During the reaction, an MoS${}_{2}$ layer is deposited on the surface of the SiO${}_{2}$/Si substrate. (**b**) Diagram showing the sequence of the photo-lithography process. (**c**) Scanning electron microscopy (SEM) of the MoS${}_{2}$ devices performed.

Devices were fabricated through the direct synthesis of large-area MoS${}_{2}$ films on an n-type silicon substrate covered by a 100 nm thick SiO${}_{2}$ layer. This substrate selection allows for the fabrication of back-gated transistors without any film transfer. Synthesis was accomplished via chemical vapor deposition (CVD) through sulfurization of molybdenum trioxide (MoO${}_{3}$) on the target substrates at a maximum temperature of 700 °C. Figure 1a (left) depicts a scheme of the process carried out in a two-temperature-zone tube furnace; a picture of the experimental setup is shown in Figure 1a (right). Further information regarding the synthesis of the MoS${}_{2}$ layer can be found in our previous work [32]. The device processing is summarized in Figure 1b: UV photo-lithography and lift-off were used to define the source and drain patterns on the MoS${}_{2}$. PMGI resist was spun at 4000 rpm for 50 s, and baked at 180 °C for 5 min, followed by spinning the S1813 resist at 4000 rpm for 50 s and baking at 115 °C for 2 min. Mask aligner was used in hard contact mode to expose the resist for 7 s. Then, the exposed resist was developed in MF-319 developer for 1 min 45 s. Electron beam evaporation was employed to deposit the Ni/Au electrodes. The thicknesses of the Ni and Au were 20 nm and 200 nm, respectively. The lift-off of the bi-layer resist stack was performed. Finally, the UV photo-lithography was repeated to perform a dry etching of the MoS${}_{2}$ material outside the channel between source and drain contacts, employing fluorine-based chemistry (SF${}_{6}$/Ar: 5/45 sccm) at a power of 100 W and pressure of 6 mT. Scanning electron microscope characterization (SEM) (TESCAN VEGA3) corroborates the well-defined processing in Figure 1c.

**Figure 2:**
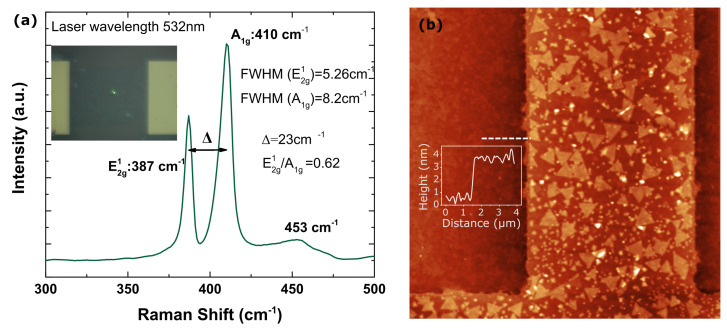
(**a**) Raman characterization of one of the MoS${}_{2}$ devices fabricated. Green dot in the inset indicates the area of characterization, corresponding with the channel of the device. (**b**) Atomic force microscopy (AFM) characterization of the channel of one MoS${}_{2}$ device. Inset presents the cross-section revealing an MoS${}_{2}$ layer thickness around 4 nm.

The chemical corroboration of the films was investigated by Raman spectroscopy (Jasco NRS-5100). Figure 2a shows the structural characterization of an MoS${}_{2}$ device (the inset indicates the channel zone where Raman characterization was performed) exhibiting the two Raman characteristic bands at 387 cm${}^{-1}$ (E${}_{2g}^{1}$) and 410 cm${}^{-1}$ (A${}_{g}^{1}$) with a separation between peaks (Δ) around 23 cm${}^{-1}$. These results are consistent with the presence of thin MoS${}_{2}$ layers [33]. Other parameters such as the full width at half maximum (FWHM) and the ratio of the peaks suggest multi-layer MoS${}_{2}$ devices [34]. Device thickness is measured using an atomic force microscope (NTMDT NTEGRA) in semicontact mode and with metallic tips. Figure 2b depicts the topography of the channel. Cross section in the inset determines around 4 nm thick MoS${}_{2}$ layer.

The electrical characteristics were measured in a Janis ST-500 cryogenic probe station with temperature, pressure and light intensity control capabilities. The direct-current (dc) characteristic was recorded using a Keithley SCS 4200 and an Agilent B1500 systems. The low-frequency noise characterization was carried out using a low-noise-current amplifier connected to a software-based spectrum analyzer [35].

## 3. Results

**Figure 3:**
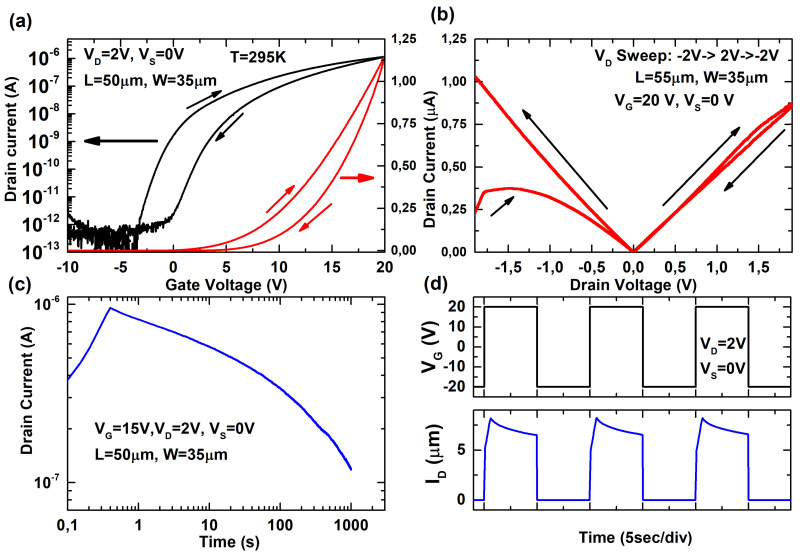
(**a**) Transfer characteristic (I${}_{D}$-V${}_{G}$) of an MoS${}_{2}$ device in logarithmic (left) and linear (right) scale. (**b**) Output characteristic (I${}_{D}$-V${}_{D}$) in a double sweep characterization. (**c**) Transient response (I${}_{D}$-t) while a positive voltage is applied at the back gate. (**d**) Transient response of the device to successive positive (stress) and reverse (recover) pulses. L = 50 μm, W = 35 μm, T = 295K at atmospheric conditions.

Figure 3a shows the transfer characteristic of a 50 μm length and 35 μm width MoS${}_{2}$ back-gated device. Left and right axes show drain current in logarithmic and linear scale, respectively. Note that a wide hysteresis is observed when a double sweep gate bias is applied. Threshold voltage is shifted to positive levels while the device is forward-biased, presenting higher channel resistance during the backward sense. This phenomenon is expected when dealing with defective or non-passivated devices [26,36]. When a forward gate bias is applied, traps whose energy are below the Fermi level are filled by electrons with different characteristic times. The trapped electrons contribute to the increase of negative charge and therefore, increase the threshold voltage. However, Figure 3b shows a less usual aspect in the output characteristic. Considerable differences are observed between the forward and the backward sweep. Particularly interesting is the initial current increase during the first seconds of the measurement. In this I${}_{D}$-V${}_{D}$ curve, one can observe a non-linear and non-symmetrical behavior between positive and negative drain biases; this effect is attributed to the Schottky barriers formation at the metal–semiconductor interface. In this regard, our previous works have determined the formation of back-to-back Schottky diodes due to Fermi level pinning at the metal–semiconductor interfaces [32,37]. This Fermi level pinning induces Schottky barriers, which affect the performance of the device, in this case, blocking the hole carriers in the transfer characteristic. These results have also been presented in the literature [38,39]. However, this Schottky barrier formation does not entirely explain the curve shape.

To clarify this aspect, Figure 3c shows the transient response of the device while a positive gate pulse is applied. As observed, there is an initial time to reach the maximum drain current, followed by a decay period with a constant current decrease. To the best of our knowledge, this initial delay in the device operation is not reported in the literature for these two-dimensional devices, while the current decay is as expected for gated devices. Note that this time-dependent response implies that device hysteresis will depend on the measuring time. Fast measurements (less than 1 s) suffer from the current increase, whereas slow measurements (more than 1 s) will suffer an initial increase and then a current decrease. Therefore, the current level and the device hysteresis in each case will be different. Another relevant aspect is the permanent or temporal characteristic of this device degradation observed while operating. Figure 3d shows the transient response of the device when successive positive (stress) and negative (recover) pulses are applied. As observed, there is a full recovery of the device characteristic after the stress period (V${}_{G}$ = 20 V) if a recover pulse (V${}_{G}$ = −20 V) is then applied. This fact points to mobile charges and trapping/detrapping phenomena as the origin of the device instability. These defects can be associated with both extrinsic (diffused water molecules and the chemisorption of oxygen) and intrinsic defect at the interface due to oxygen and sulfur vacancies [40,41,42], or even defects due to a non-passivated surface bond of the MoS${}_{2}$ [43]. Otherwise, in case of traps generation during the stress process (as observed in some devices due to bias temperature instability), the device would present a permanent degradation and the current level will not be fully recovered after the reverse bias pulse. On the other hand, the initial time to reach the maximum inversion charge is not extensively observed. In our previous work, we carried out an initial study in this regard, attributing this phenomenon to substrate limitations to reach the inversion layer at the back interface (SiO${}_{2}$/Si) due to the low doping level [32]. This limitation could be mitigated by peripheral inversion, a coupling effect, which usually happens in semiconductor devices when leaving regions exposed to ambient conditions or when there are residual charge on oxide surfaces [44]. If the density of the residual charge is higher than the semiconductor charge associated with the maximum depletion width, this charge may result in the depletion of the semiconductor surface and the generation and recombination of minority carriers. Despite the fact that this phenomenon explains the initial delay to reach the inversion charge in the devices, extensive characterization under different environmental conditions will reveal how to mitigate or enhance this for sensing applications.

**Figure 4:**
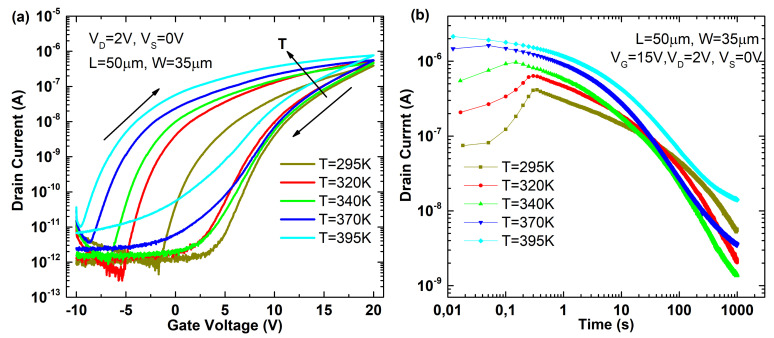
(**a**) Transfer characteristic (I${}_{D}$-V${}_{G}$) of an MoS${}_{2}$ device for different temperatures. (**b**) Transient response (I${}_{D}$-t) for different temperatures when a positive voltage is applied at the back gate.

Figure 4 shows the transfer characteristic (Figure 4a) and the transient response (Figure 4b) of an MoS${}_{2}$ device when increasing the temperature. Transfer characteristic reveals both a reduction of the threshold voltage and an increase of the hysteresis in the double sweep measurement. The threshold voltage shift can be explained by the higher thermal generation and the subsequent carrier density increase. Hysteresis signature can be derived from Figure 4b. On the one hand, the initial phenomenon implies an increase of the drain current to reach the maximum inversion charge, which then decreases at higher temperatures. This implies that the device can respond faster and points to carrier concentration limitations (lower concentration at lower temperature) as the origin of this initial phenomenon. On the other hand, the current decay, governed by carrier trapping phenomena and mobile ions, also increases with the temperature. This current degradation increasing with temperature is usually attributed to an enhancement of carrier trapping/detrapping events known as bias temperature instabilities (BTI) and is mainly attributed to process-related pre-existing defects or interface trap generation, whose time dynamics are described by the reaction–diffusion (RD) model [45,46].

**Figure 5:**
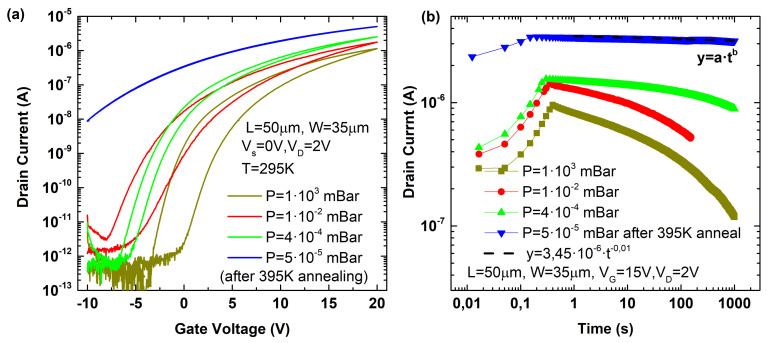
(**a**) Transfer characteristic (I${}_{D}$-V${}_{G}$) of an MoS${}_{2}$ device for different pressure conditions. (**b**) Transient response (I${}_{D}$-t) for different pressure conditions when a positive voltage is applied at the back gate.

Figure 5 shows the transfer (Figure 5a) and transient (Figure 5b) characteristics of the device when different pressure conditions are set, from atmospheric pressure up to high vacuum. Lower pressure (higher vacuum) improves the behavior of the device, reducing the current decay, which also implies a narrower hysteresis. The increase of the current level at low pressures suggests a decrease of the threshold voltage and therefore a defect charge density diminution (less ions or charged defects). Especially relevant is the case of high vacuum after an annealing period at high temperature (395 K)—marked by the blue symbols. The hysteresis (Figure 5a) and current degradation (Figure 5b) for this case are almost negligible. This result suggests that mobile ions and charges are mainly due to adsorbates and ambient contaminants, which can be removed at high temperature, when vacuum conditions limit their reappearance. After the removal of adsorbates, the current decay follows a power–law time dependence attributed to trapping/detrapping mechanisms in pre-existing traps (intrinsic traps) [45]. Note also that this result is in agreement with the higher degradation observed at high temperature and atmospheric conditions (Figure 4) due to the presence of contaminants together with the trapping/detrapping mechanisms enhanced by the bias temperature instability. However, the impact of the pressure on the initial time to reach the maximum current level is minimal when compared to the temperature correlation observed in Figure 4. This means that the initial time to reach the maximum inversion charge is independent of these contaminants and adsorbates.

**Figure 6:**
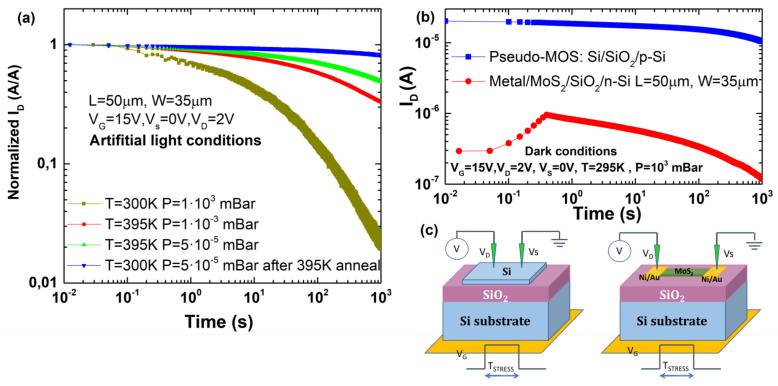
(**a**) Transient response (I${}_{D}$-t) for different pressure conditions under artificial light conditions when a positive voltage is applied at the back gate. (**b**) Transient response (I${}_{D}$-t) comparison between a silicon pseudo-MOS device and the MoS${}_{2}$ back-gated transistor at atmospheric and dark conditions. (**c**) Scheme of the pseudo-MOS (**left**) and the MoS${}_{2}$ device (**right**) measurement configuration.

Contrarily to the previously observed, if an artificial light source (wide-spectrum controlled intensity lamp) is employed to illuminate the device during the characterization, it does not show the initial ramp to reach the maximum current, as Figure 6a shows. A change in light source could mean an increase of the carrier density; therefore, the current delay experience under dark conditions can be related to an initial carrier limitation. Different effects can be considered to explain this limitation. Considering that the silicon substrate is not highly doped (N${}_{D}$∼ 10${}^{16}$cm ${}^{-3}$), inversion at the back interface depends on the thermal generation/recombination of minority carriers. This slow process at the back interface may suppose an initial lack of majority carriers at the front interface. Illumination (or high temperature) can accelerate the carrier generation (thermal generation) decreasing this delay time. Moreover, the minority carrier density at the back interface can also be affected by channel or surface pre-existent charges through peripheral inversion effect. Fixed charges in the channel or at interface can induce an opposite-sign charge at the back interface, facilitating or inhibiting the inversion layer formation (depending on the sign). Additionally, other effects can also contribute to the initial current delay. Due to the high Schottky barriers induced by the Fermi level pinning, holes carriers, i.e., minorities considering n-type semiconductor, cannot easily escape from the channel through the contacts. This charge can have a twofold effect. Positive charge at the front interface may induce a negative charge at the back interface by peripheral inversion, limiting the layer inversion and it may enhance the carrier recombination reducing the majority carrier density.

To shed light on which of these mechanisms can have more influence on the origin of this initial current delay in the transient response, devices with a similar substrate structure, but different active layer technology, were tested. Figure 6b compares the transient response of the devices for two different technologies at room temperature and atmospheric conditions: (i) the one we characterized previously, a 55 μm length and 35 μm width MoS${}_{2}$ device with two Ni/Au contacts as source and drain and a SiO${}_{2}$/low n-type doped silicon layer acting as back gate, with (ii) a pseudo-MOS transistor and a p-type active silicon layer on a SiO${}_{2}$/low p-type doped silicon layer acting as back gate. A scheme of the two different technologies is shown in Figure 6c. The main similarity between these devices is the use of low doped layers as channel and back gate terminals, and the absence of a passivation layer in the device surface (bare devices). The main differences are: (i) the active layer material (MoS${}_{2}$ vs. low doped Si) and (ii) the transport mechanism at the metal/semiconductor interface, which displays ohmic behavior due to tunneling from the needles to the channel in the case of the pseudo-MOS transistor [47] versus thermo-ionic emission due to Schottky barrier formation in MoS${}_{2}$ devices [36]. As observed in Figure 6b, the pseudo-MOS device is not suffering from the initial delay to form the inversion charge. Despite the fact that this result cannot totally discard an isolated effect of the substrate as the origin of the initial instability (because the devices are quite technologically different), it points to an effect induced by the singular characteristics of the MoS${}_{2}$ active layer more than an effect due to the low doped substrate structure. Possible explanations to this effect could be pre-existent intrinsic charges (charged defects or minority carriers) in the channel, which induce inversion limitations at the back interface (by peripheral inversion) or/and initial recombination of majority carriers in the channel (reducing the active carrier density) or/and current delay enhanced by the large Schottky barriers at the contacts.

## 4. Discussion and Application

**Figure 7:**
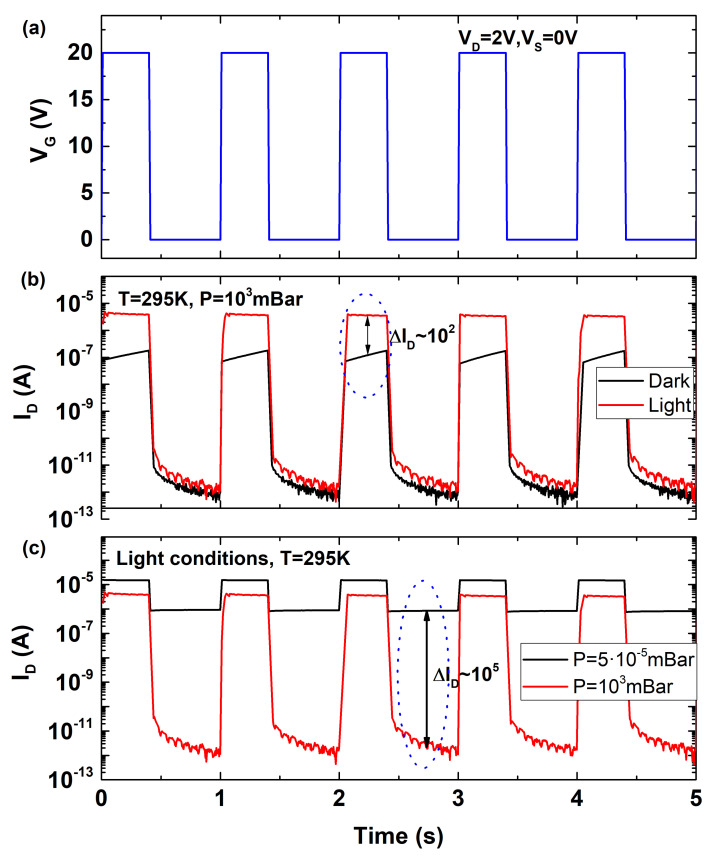
(**a**) Successive back-gate pulsed biasing pattern. (**b**) Drain response of the device at atmospheric pressure and room temperature for illuminated and dark conditions. (**c**) Drain response at room temperature and light conditions for low and atmospheric pressure.

We determined the effect of the disturbing mechanisms on the transient response and the hysteresis of the MoS${}_{2}$ devices so that they can be exploited for sensing applications. The slow initial response of the device seems to be due to the singular characteristics of the CVD-grown MoS${}_{2}$ layer. Light or high temperature conditions alleviate it while mobile charge densities (due to adsorption) do not affect this initial limitation in the device response. This phenomenon can be exploited for light sensing as the device will present a slow response (temporally lower current) under dark conditions compared to light conditions. Figure 7 shows the device response to successive short gate voltage pulses. Figure 7a depicts the bias pattern. Figure 7b shows the response of the device under light and dark conditions. As highlighted by the dashed circle, the current operation at high voltage is around two orders of magnitude higher for the case of illuminated device. Note that this current difference is enhanced by the slow reaction of the device to dark conditions. As previously demonstrated, these devices also present a threshold voltage shift depending on pressure and temperature conditions. Absence of adsorbates and mobile ions reduces the drain current degradation reducing the threshold voltage as well. At fixed temperature, this effect can be exploited for pressure sensing. Figure 7c shows the response of the device under light conditions at room temperature under atmospheric pressure and high vacuum conditions. Due to the high carrier density induced by illumination, on-state current is similar for both pressures (fast response), however, the current decay and the threshold voltage are lower at vacuum conditions (less density of mobile ions), which means a much higher current level when the device is biased closer to V${}_{G}$ = 0 V. This effect can be exploited to sense pressure by comparing the current level (dashed line). Another advantage is that, under controlled temperature conditions, both sensing capabilities, light and pressure can be simultaneously exploited. Comparing the current level at high gate voltages can determine the light/dark condition while differences at low gate bias can determine the pressure conditions. These results reflect that these devices can be employed as cheap light and pressure sensors without the need of complex passivation processes by just taking advantage of the inherit operational limitations.

## 5. Conclusions

In this work, the electrical hysteresis and the transient response of as-synthesized MoS${}_{2}$ back-gated devices have been extensively analyzed. Hysteresis is governed by different degrading mechanisms. The transient response of the device determines that there is an initial delay to achieve the maximum inversion layer charge. This is mitigated at high temperature or under light conditions, pointing to lack of initial carriers as the possible origin. This effect can be exploited to design a light sensor detecting the drain current level differences. Additionally, the devices suffer from time-dependent current decay, which is induced mainly by mobile charges (adsorbates) and by carrier trapping/detrapping mechanisms. However, at low pressure conditions, the density of these mobile ions is reduced, decreasing the threshold voltage of the device and allowing for the easy detection of pressure conditions by comparing the current level at low gate voltages. Interestingly, both sensing capabilities can be used simultaneously.
